# Automated Volumetric Milling Area Planning for Acoustic Neuroma Surgery via Evolutionary Multi-Objective Optimization

**DOI:** 10.3390/s25020448

**Published:** 2025-01-14

**Authors:** Sheng Yang, Haowei Li, Peihai Zhang, Wenqing Yan, Zhe Zhao, Hui Ding, Guangzhi Wang

**Affiliations:** 1 School of Biomedical Engineering, Tsinghua University, Shuang Qing Road, Beijing 100084, China; yangs19@mails.tsinghua.edu.cn (S.Y.);; 2 Department of Neurosurgery, Beijing Tsinghua Changgung Hospital, Li Tang Road, Beijing 100043, China; 3 School of Clinical Medicine, Tsinghua University, Shuang Qing Road, Beijing 100084, China; 4 Orthopedics & Sports Medicine Center, Beijing Tsinghua Changgung Hospital, Li Tang Road, Beijing 100043, China

**Keywords:** volumetric surgical planning, volumetric area parameterization, multi-objective optimization, bone milling

## Abstract

Mastoidectomy is critical in acoustic neuroma surgery, where precise planning of the bone milling area is essential for surgical navigation. The complexity of representing the irregular volumetric area and the presence of high-risk structures (e.g., blood vessels and nerves) complicate this task. In order to determine the bone area to mill using preoperative CT images automatically, we propose an automated planning method using evolutionary multi-objective optimization for safer and more efficient milling plans. High-resolution segmentation of the adjacent risk structures is performed on preoperative CT images with a template-based approach. The maximum milling area is defined based on constraints from the risk structures and tool dimensions. Deformation fields are used to simplify the volumetric area into limited continuous parameters suitable for optimization. Finally, a multi-objective optimization algorithm is used to achieve a Pareto-optimal design. Compared with manual planning on six volumes, our method reduced the potential damage to the scala vestibuli by 29.8%, improved the milling boundary smoothness by 78.3%, and increased target accessibility by 26.4%. Assessment by surgeons confirmed the clinical feasibility of the generated plans. In summary, this study presents a parameterization approach to irregular volumetric regions, enabling automated milling area planning through optimization techniques that ensure safety and feasibility. This method is also adaptable to various volumetric planning scenarios.

## 1. Introduction

Establishing a surgical channel is a critical step in acoustic neuroma surgery. During this procedure, a portion of the skull must be removed via milling or drilling to expose the surgical target for subsequent interventions [1]. The morphology of the removed skull area significantly impacts the surgeon’s ability to access the target region effectively in later stages of the operation. Additionally, the proximity of the milling site to the surrounding structures and the volume of bone to be excised are crucial factors in assessing potential postoperative complications. Consequently, preoperative planning of milling areas enables surgeons to gain a comprehensive understanding of the surgical procedure and assess the feasibility of the intervention, thereby promoting successful operations with minimal risk and potential harm [2].

A surgical channel refers to the space created by milling part of the temporal bone, thereby exposing intracranial lesions. Robotic surgery for mastoidectomy has been developed to enhance the stability and precision in creating surgical channels [3,4,5,6]. Additional surgical navigation techniques, such as image-guided surgery and augmented reality navigation, can further assist with the bone milling procedure [7,8,9,10]. For these navigation techniques, preoperative milling area planning is increasingly crucial, serving as a spatial reference to guide milling operations. However, planning such a volumetric area is challenging due to the complexity of its spatial representation and the constraints imposed by the surrounding structures. For instance, the translabyrinthine approach is a transpetrosal presigmoid surgical route used for operative treatment. It is employed to quickly access the cochleovestibular nerve and treat tumors located in the lateral skull base [11]. In this approach, the surgical channel must navigate around critical vessels and nerves [1,12], damage to which could lead to severe complications such as cerebrospinal fluid leaks and facial nerve dysfunction [13,14]. The current automated planning algorithms under such constraints predominantly address cochlear implants [15] and stereoelectroencephalography (SEEG) [16], where only a linear trajectory is required, leaving the planning of complex volumetric areas an unresolved issue. Practically, preoperative planning of milling areas largely depends on manual planning by surgeons, which takes 35 minutes on average [17] and often lacks comprehensive consideration of the constructability of the planned milling area and the subsequent surgical procedures [4,5], limiting the effectiveness of preoperative planning in acoustic neuroma surgeries.

To address the aforementioned challenges, this study proposes a set of methods for modeling volumetric milling areas and the surrounding spatial constraints to enable automatic optimization. We aim to achieve automated planning for milling areas while ensuring the safety, constructability, and accessibility of the surgical targets in subsequent operations. We first employ high-resolution local segmentation masks of the key surrounding structures and deformable registration to construct the spatial distribution of critical structures, serving as constraints for subsequent planning algorithms. By combining the spatial constraints imposed by key structures with the physical dimensions of the surgical tools, the maximum milling area is generated as the initial input for optimization. Subsequently, a series of deformation fields are designed to control the volumetric milling area, using a surgical target accessing procedure as the foundational model. This method simplifies the complex three-dimensional volumetric area into a set of continuous parameters, while the boundaries formed by adjacent structures are converted into numerical constraints on these parameters. Finally, target accessibility, potential risk of injury, and the constructability of the surgical channel are quantitatively evaluated, with an evolutionary multi-objective optimization algorithm applied to automatically generate a surgical plan with optimal safety and feasibility. In detail, our method can generate a surgical plan in 5840.1 ± 279.9 s, reducing potential damage to the scala vestibuli by 29.8%, improving the milling boundary smoothness by 78.3%, and increasing target accessibility by 26.4%.

This paper makes the following main contributions:

- A set of spatial modeling methods for parameterizing irregular volumetric milling areas;
- Evaluation methods for milling areas that are critical to the constructability of the surgical channel;
- The first automated planning algorithm which enables volumetric spatial planning within a feasible amount of time.

## 2. Related Work

Currently, most surgical planning studies in neurosurgery emphasize trajectory planning, such as electrode insertion for deep brain stimulation [18] and inner ear access for cochlear implantation [15,19,20]. In these cases, the planning target can be simply represented by an entry point on the skull and a target point within the brain. The representation limits the target definition space and allows each trajectory to be straightforwardly evaluated, thereby facilitating the application of optimization algorithms to these planning problems. In contrast, defining an irregular volumetric area presents a far more complex challenge. The inability to accurately model the planning target not only makes the problem unsuitable for automatic optimization but also complicates the evaluation of the planned surgical channel. Therefore, the current research primarily employs heuristic methods to generate milling area plans, avoiding the need to explicitly parameterize an irregular volume. Popovic et al. [21] simplified this problem by neglecting the thickness of the skull, allowing them to project the three-dimensional contours of a tumor directly onto the adjacent skull surface to define the resection area. However, this method is unsuitable for temporal regions, where the bone thickness varies considerably, while critical structures, such as the vessels and nerves, are present. In a different approach, McBrayer et al. [17] pre-planned surgical channels on an atlas with segmented critical structures. After that, deformable volume registration was applied to map the surgical plan from the atlas onto the new CT data. However, this method overlooks how the planned milling area impacts the constructability of the surgical channel and subsequent surgical procedures. Although the milling area may be well planned in the atlas, the deformation process can reduce its feasibility in real-world operations. Aghdasi et al. [22] and Rajesh et al. [2] enhance this method by creating multiple plans on the atlas. Unlike McBrayer et al. [17]’s study, segmentation of the critical structures was also performed on the new CT data, with each milling plan evaluated in relation to the surrounding structures to identify the least risky option. Nonetheless, these approaches still lack personalized consideration of the surgical targets and a quantitative assessment of the plan’s feasibility.

## 3. Methods

### 3.1. Problem Definition

**Figure 1:**
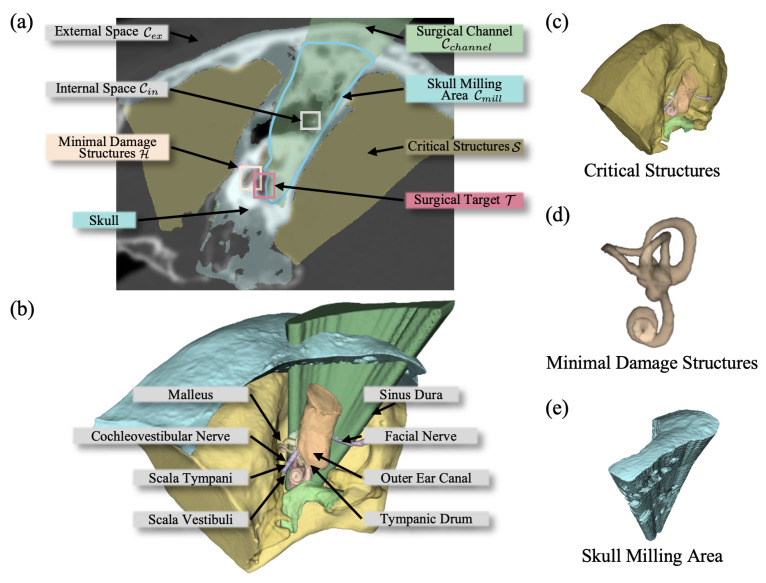
(**a**,**b**) Spatial relationship between the skull, milling plan, and surrounding structures. (**c**) Critical structures that should not be damaged during surgery. (**d**) Structures that should be damaged as little as possible. (**e**) Planned milling area.

**Planning target:** As illustrated in Figure 1, surgeons must remove part of the skull $C_{mill}$ to expose the surgical target T during acoustic neuroma surgery. This removal creates the surgical channel $C_{channel}$, composed of the milled skull, the internal cavities $C_{in}$, and external space $C_{ex}$, defined as $C_{channel}=C_{ex}\cup C_{in}\cup C_{mill}$.

**Spatial constraints:** While the skull forms the definition space for milling operations, the surrounding critical structures impose certain limits on this area. Based on their clinical significance, the surrounding structures can be categorized into those that must remain intact $H=\cup_{i=1}^{I}C_{h}^{i}$, such as the sinus dura and the facial nerve, and those that should be minimally damaged $S=\cup_{j=1}^{J}C_{s}^{j}$, such as the scala vestibuli.

**Evaluation:** During the planning of the milling area, the potential risks $Q_{k}\left(C_{mill}\right),k\in K$ relevant to the design of the area need to be considered. Key evaluations include the difficulty of constructing the planned surgical channel $Q_{c}$, how difficult it is for the surgical tools to reach the surgery targets after bone removal $Q_{a}$, and how much injury to the structures S is caused by the milling procedure $Q_{i}$.

The comprehensive planning problem can thus be formulated as optimizing the milling area $C_{mill}$ while ensuring it does not intersect with critical structures $C_{mill}\cap C_{h}^{i}=⌀$, aiming to minimize surgical risks $Q_{k}\left(C_{mill}\right),k=1,\ldots ,K$.

### 3.2. The Automated Milling Area Planning Pipeline

**Figure 2:**
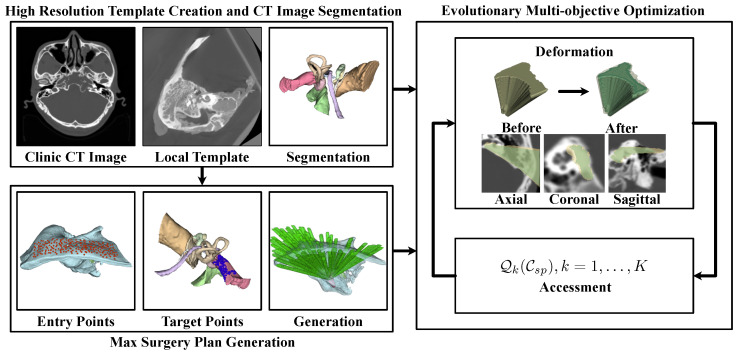
Automatic milling area planning pipeline.

However, the extremely high complexity of defining volumetric milling areas can result in impractical optimization times. To address this, we propose the framework illustrated in Figure 2 for automated planning. Initially, a template-based approach was employed to segment the high-resolution risk structures S and H surrounding the milling areas. Subsequently, potential entry points on the skull surface K and target points T on the surgical target were identified.

For each target t∈T, the maximum feasible milling area $C_{mill}^{\left(0\right)}\left(t\right)$ that contributes to the surgical procedure was calculated, adhering to the constraints of critical structures H. This process generated a maximum potential milling region, which served as the boundary for optimization: $C_{mill}^{\left(0\right)}=\cup_{t\in T}C_{mill}^{\left(0\right)}\left(t\right)$.

To control these volumetric areas, a set of deformation control functions $h_{t}\left(p_{i}^{t}\right)$ was designed. By projecting each $C_{mill}^{\left(0\right)}\left(t\right)$ onto a control plane and applying $h_{t}$, the volumetric milling area could be modulated using the parameters defined by these functions. This approach inherently transformed the complex spatial constraints into numerical boundaries for parameter adjustments. Lastly, we evaluated patient injury $Q_{i}$, constructability $Q_{c}$, and target accessibility $Q_{a}$ for the surgical channel under the parameter set $P=p_{i}^{t}$. Multi-objective optimization was then employed to identify the most suitable surgical plan.

(1) $$\begin{array}{cc} \; & C_{mill}=\underset{t\in T}{⋃}h_{t}\left(C_{mill}^{\left(0\right)}\left(t\right)\right)\\ \min\; & Q_{c}\left(C_{mill}\right),\;Q_{a}\left(C_{mill}\right),\;Q_{i}\left(C_{mill}\right)\\ s.t.\; & P=\{p_{i}^{t}\in \left(0,1\right],\;i=1,\ldots ,I,\;\;t=1,\ldots ,T\} \end{array}$$

The following part of this paper details the methods proposed in this paper. In detail, Section 3.3 includes the method for critical structure segmentation, Section 3.4.1 includes the method for generating the maximum possible milling area, Section 3.4.2 includes the method for volumetric milling area parameterization, and Section 3.4.3 includes the milling area evaluations and the implementation of multi-objective optimization, while Section 3.5 includes a set of methods for computing acceleration to enable automatic planning within a reasonable amount of time.

### 3.3. Critical Structure Segmentation

Segmentation of the critical structures, such as the sinus dura, the facial nerve, and the scala tympani, surrounding the milling area in preoperative CT scans is essential for establishing the spatial constraints that inform the milling area’s design. However, this segmentation task is challenging due to several factors: the dimensions of certain critical structures approach the resolution limit of clinical CT devices (0.625 mm), soft tissues can be difficult to discern in CT images, and there is considerable variation in the sizes of different critical structures. These complexities pose significant challenges for deep-learning-based segmentation methods [23,24,25].

**Figure 3:**
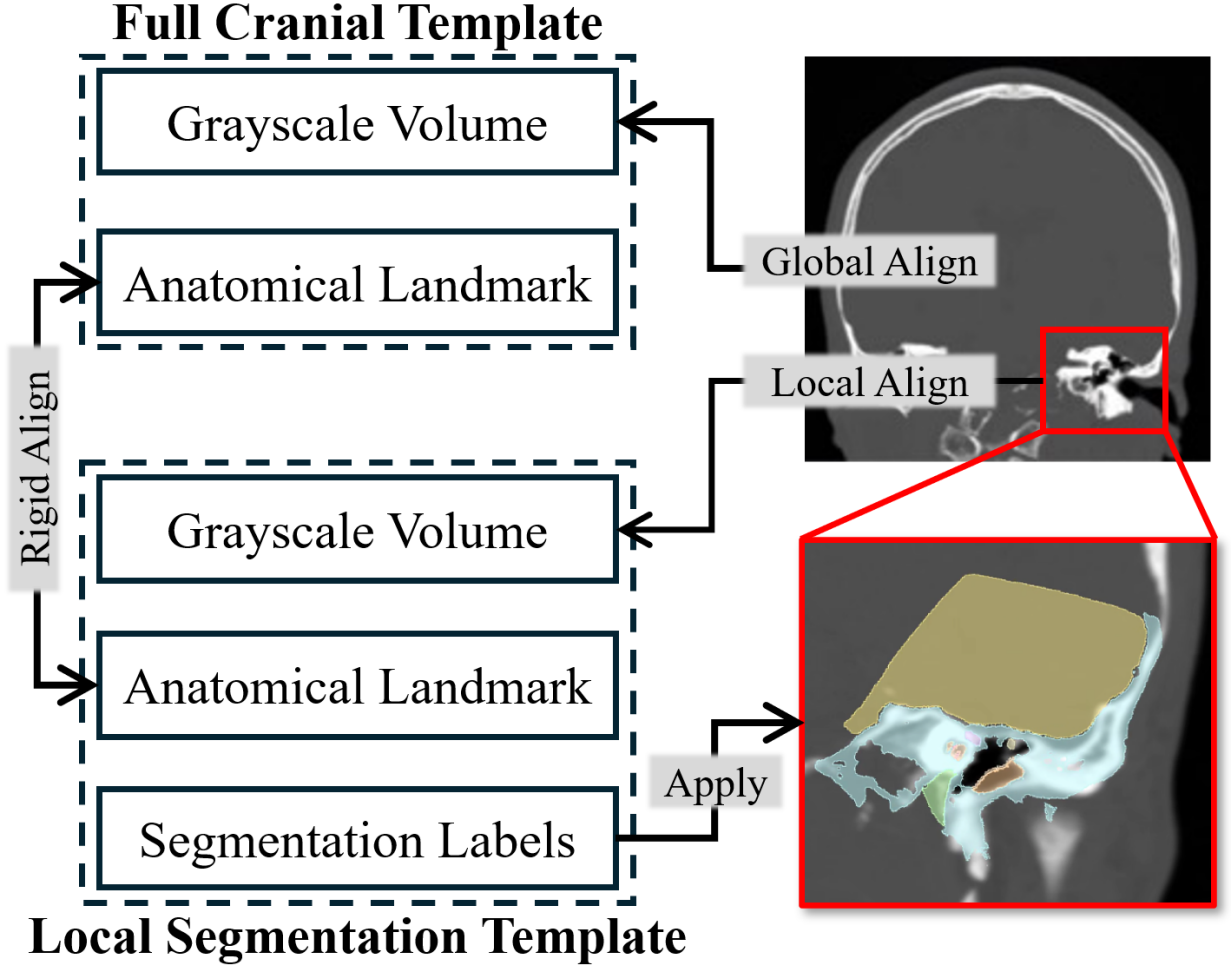
Template-based critical structure segmentation. Global template registration for initial alignment between the local template and the new CT volume. Local template registration for high-resolution segmentation.

**Figure 4:**
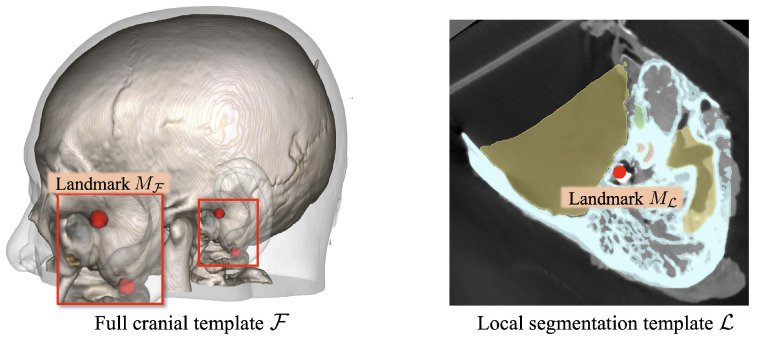
Full cranial template and local segmentation template for segmentation of the critical structures surrounding the milling area.

Therefore, we employed the template-based segmentation pipeline illustrated in Figure 3 to achieve robust segmentation of the critical structures beyond the imaging resolution limitations. Specifically, two cranial CT templates were developed (see Figure 4): one encompassing the full cranial area, F, at a resolution similar to that used in standard clinical practice (0.625 mm × 0.625 mm × 0.625 mm), and another covering only the local region, L, around the lateral skull base area with a finer spatial resolution (0.125 mm × 0.125 mm × 0.125 mm). The full cranial template F served to establish the spatial connection between the target CT volumes and the high-resolution local template. To enhance the generalizability of template F, F was generated by registering and averaging multiple clinical CT volumes using the deformable registration method proposed in Avants et al. [26]’s study. Mutual information (MI) was employed as the optimization metric during the registration process. Concurrently, the local template L was designed to provide a precise reference for accurate segmentation. To accomplish this, we utilized the high-resolution temporal bone segmentation dataset from Sieber et al. [27]’s study, averaging it to form L. Moreover, to connect these two different spaces, anatomical landmarks, including the stylomastoid foramen, the geniculate ganglion, the top of the head of the malleus, the footplate of the stapes, and the arcuate eminence, were annotated on both templates, represented as $M_{F}$ and $M_{L}$.

For segmentation of a new CT volume V, it was first aligned with the full-head template F through deformable registration to transfer the anatomical landmarks into the new volume space, namely $M_{V}$. Subsequently, rigid body registration between $M_{L}$ and $M_{V}$ was performed using Singular Value Decomposition (SVD) to achieve an initial alignment between L and V. This was followed by deformable registration between the CT volumes in L and V, generating the non-rigid transformation D mapping the high-resolution template L to V. Finally, the deformation field was applied to transfer the segmentation labels $L_{seg}$ into the new volume space $D(L_{seg})$, generating the spatial constraints H,S for subsequent planning. This approach ensures that the segmented critical structures retain the high resolution of the local template, while structural integrity and smoothness are maintained through the deformation-based method.

### 3.4. Automated Milling Area Planning

#### 3.4.1. Maximum Milling Area Generation

To define a boundary for milling area planning and establish a foundation for quantifying the spatial constraints posed by critical structures, a maximum permissible milling area was first generated based on the structures that must remain intact during surgery H. Prior to generating this area, the surgical target T was manually annotated, while potential entry points K were automatically identified from the skull surface near the surgical target.

For the construction of a feasible surgical channel, each milled voxel $s\in C_{mill}$ should contribute to the surgical procedures following channel creation. Simplifying this by modeling the surgical tool as a cylinder, *s* should meet the condition that an entry point k∈K and a target point t∈T exist such that the cylindrical region connecting *k* and *t* with the radius *r* encompasses *s*:

(2) d(s,l(k,s))<r

where *l* denotes the linear trajectory between *k* and *t*, and d(s,l) represents the distance from the spatial point *s* to the trajectory *l*.

Consequently, the maximum permissible milling area can be heuristically represented as the union of all potential trajectories of the surgical tools that avoid interaction with the critical structures:

(3) $$C_{mill}^{\left(0\right)}=\underset{k\in K,t\in T}{⋃}I\left(k,t\right)\;\cdot \;C\left(k,t\right)$$

Here, I(k,t) represents whether the surgical tool will damage any critical structure when accessing *t* though *s*:

(4) $$I\left(k,t\right)=\left\{\begin{array}{c} 1\;d\left(p_{n}^{i},l\left(k,t\right)\right)>r+d_{risk}^{i},\;\forall p_{n}^{i}\in C_{h}^{i},\;\forall i\\ 0\;else \end{array}\right.$$

where $d_{risk}^{i}$ denotes the empirically minimum safe distance that the surgical tools should maintain from each critical structure. In our implementation, a safe distance of 0.5 mm was enforced for the scala tympani, the scala vestibuli, the malleus, the incus, the stapes, the chorda tympani, the tympanic drum, and the carotis, while 1.5 mm was set for the sinus dura, and 2.5 mm was set for the facial nerve.

Meanwhile, C(k,t) in Equation (3) represents the cylindrical spatial area covered by the surgical tool:

(5) $$C\left(k,t\right)=\{s|\;d\left(s,l\left(k,t\right)\right)<r,\;\vec{ks}\;\cdot \;\vec{kt}>0,\;\vec{ts}\;\cdot \;\vec{tk}>0\}$$

Although this method effectively generates the maximum permissible milling area while ensuring the validity of each milled voxel, the volumetric region is controlled by a set of unordered point pairs, {(k,t)}, which is unsuitable for further refinement. Therefore, we categorize {(k,t)} further based on each spatial element *t* within the surgical target:

(6) $$C_{mill}^{\left(0\right)}=\underset{t\in T}{⋃}C_{mill}^{\left(0\right)}\left(t\right),\;C_{mill}^{\left(0\right)}\left(t\right)=\underset{k\in K}{⋃}I\left(k,t\right)\;\cdot \;C\left(k,t\right)$$

Because the critical structures are continuous, all feasible entry points *k* for a single target form a continuous distribution K(t) on the skull surface. Therefore, the maximum milling area can be expressed as the set of each spatial element in the surgical target paired with all feasible entry points relevant to that target, namely {(t,K(t))∣t∈T}. The continuity of K(t) makes it eligible for parameterization.

Moreover, to ensure accuracy in milling area planning, the preoperative CT scans, critical structure segmentations, entry points, and target points were uniformly resampled to a spatial resolution of 0.25 mm prior to maximum milling area generation.

#### 3.4.2. Volumetric Area Parameterization

The previous generation of the maximum milling area organized the irregular volumetric region into target points and corresponding feasible entry points for each target, represented by {(t,K(t))∣t∈T}. However, the irregular skull surface on which K(t) is distributed presents challenges for complete parameterization.

To address this issue, we selected a control plane S parallel to the approach direction of the surgical operations (the A-S plane in RAS space). Each connection between *t* and *k*, where k∈K(t), was then extended along the vector $\vec{tk}$ to intersect with S at point *g*. The generated point *g* then serves as a unique substitute for *k*, transforming the irregular distribution on the skull surface into a function for area selection $B_{t,S}$ on the plane S:

(7) $$B_{t,S}\left(x,y\right)=1\Leftrightarrow I\left(S\left(x,y\right),t_{n}\right)>0$$

where s(x,y) represents the 3D spatial position of a point (x,y) on the control plane.

Since K(t) is continuously distributed over the skull surface, the corresponding $B_{t,S}\left(x,y\right)$ forms a single connected component on the control plane. This property ensures that each point *k* within a subregion $B^{\prime }$ of $B_{t,S}$ ($B^{\prime }|\;B^{\prime }\left(x,y\right)<B_{t,S}\left(x,y\right),\;\forall x,y$) meets the spatial constraints imposed by structure H.

**Figure 5:**
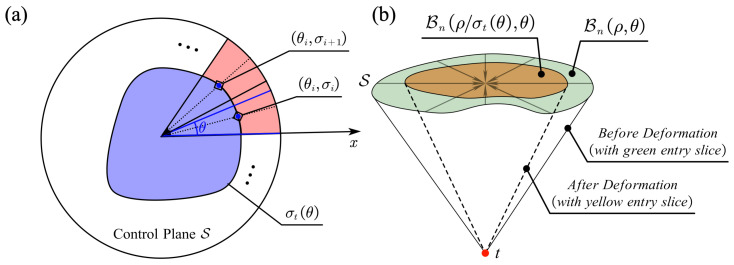
(**a**) Parameterized controlling function for milling area deformation. (**b**) Milling area before and after deformation at each slice.

To regulate the area $B_{t,S}$ for optimization purposes, we developed a control function to transform $B_{t,S}$ into a subset. For convenience, $B_{t,S}\left(x,y\right)$ was initially converted into the polar coordinate form $B_{t,S}\left(\rho ,\theta \right)$, centered at its mass center. Subsequently, a control function $\sigma_{t}\left(\theta \right),\;\theta \in \left(0,2\pi \right],\;\sigma_{t}\left(\theta \right)\in \left(0,1\right]$ was applied to modify $B_{t,S}\left(\rho ,\theta \right)$ (see Figure 5):

(8) $$h_{t}\left(B_{t,S}\right)\left(\rho ,\theta \right)=B_{t,S}\left(\rho /\sigma_{t}\left(\theta \right),\theta \right)$$

where h(·) represents the deformation operation. Given the distribution of the critical structures, the shapes of $B_{t,S}$ are predominantly convex. Hence, the deformed area $h(B_{t,S})$ generally remains a subset of $B_{t,S}$ in most cases. Nevertheless, a subset restriction was applied to the deformation process to ensure adherence to the spatial constraints:

(9) $$h_{t}^{\prime }\left(B_{t,S}\right)\left(\rho ,\theta \right)=B_{t,S}\left(\rho /\sigma_{t}\left(\theta \right),\theta \right)\;\cdot \;B_{t,S}\left(\rho ,\theta \right)$$

Finally, the proposed function $\sigma_{t}\left(\theta \right)$ was parameterized using *N* control points $\left\{{\left(\theta_{i},\sigma_{i}\right)}_{t}|\theta_{i}=i/N\;\cdot \;2\pi \right\}$ uniformly distributed along θ. The value of $\sigma_{t}$ at each θ was then determined through spline interpolation of the surrounding control points and constrained to the range (0,1]. This approach allows for complete parameterization of the milling area:

(10) $$C_{mill}^{\left(opt\right)}\left(\left\{{\left(\theta_{i},\rho_{i}\right)}_{t}\right\}\right)=\underset{t\in T}{⋃}\underset{k\in S}{⋃}B_{t,S}\left(\rho /\sigma_{t}\left(\theta \right),\theta \right)\;\cdot \;C\left(k\left(g\right),t\right)$$

In this way, the entire volumetric area can be fully governed by the parameter set $\left\{{\left(\theta_{i},\rho_{i}\right)}_{t}\right\}$, consisting of $N\;\cdot \;N_{T}$ parameters within the range (0,1], with an initial value of 1 for optimization. Here, *N* denotes the number of control points for a single control function, and $N_{T}$ represents the number of target points considered during optimization. Furthermore, the proposed modeling method, along with initialization using the maximum milling area, transforms the complex spatial constraints imposed by the surrounding critical structures into a numerical constraint on the parameters.

#### 3.4.3. Milling Area Evaluation and Optimization

To achieve the optimal milling plan, we implemented a series of quantitative assessments, evaluating the feasibility of surgical channels at different parameter settings, including the accessibility of the surgical target after channel construction $Q_{a}$, the potential injury to bone and the surrounding structures $Q_{i}$, and the complexity of constructing the surgical channel $Q_{c}$.

**Surgery target accessibility $Q_{a}$:** In Mo [28]’s study, a series of evaluation methods for surgical channel construction was proposed, including an intrinsic measure of the spatial accessibility $\mu_{a}\left(C\right)$:

(11) $$\mu_{a}\left(C\right)=\frac{1}{V\left(T\right)}\sum_{\delta t\in T}A_{\delta t}\left(C\right)$$

Here, $A_{\delta t}\left(C\right)$ represents the difficulty of accessing a specific target δt through the constructed surgical channel C [28]. Therefore, we adopted this as the metric for surgical target accessibility:

(12) $$Q_{a}\left(C_{mill}\right)=-\mu_{a}\left(C_{mill}\right)$$

**Injury $Q_{i}$:** During surgical channel construction, minimizing the damage to both the skull and the surrounding critical structures S is crucial. Therefore, the proportion of damaged volume within each tissue was adopted as a measure to evaluate the injury sustained during bone milling:

(13) $$Q_{i}\left(C_{mill}\right)=\lambda_{bone}\;\cdot \;\frac{V\left(C_{mill}\right)}{V\left(C_{bone}\right)}+\sum_{j=1}^{J}\lambda_{j}\;\cdot \;\frac{V\left(C_{mill}\cap C_{s}^{j}\right)}{V\left(C_{s}^{j}\right)},\;\lambda_{bone}+\sum_{j=1}^{J}\lambda_{j}=1$$

where $\lambda_{j}$ and $\lambda_{bone}$ denote the weights assigned to the injury of different critical structures.

**Surgical channel constructability $Q_{c}$:** In Mo [28]’s study, a metric was also proposed for evaluating the global spatial compactness $\mu_{gsc}\left(C\right)$ of the constructed surgical channel:

(14) $$\mu_{gsc}\left(C\right)=\sum_{\delta a\in C}\sum_{\delta b\in C}\left(\frac{1}{d_{\delta a,\delta b}}\right),\delta a\neq \delta b$$

where δa and δb represent distinct spatial elements within the surgical channel C, and $d_{\delta a,\delta b}$ is the Euclidean distance between these locations. If an obstacle exists between δa and δb, $d_{\delta a,\delta b}$ is set to *∞*. Generally, $\mu_{gsc}\left(C\right)$ indicates the compactness of the surgical channel. A more compact channel offers greater flexibility for adjusting the milling tool’s orientation during construction, facilitating easier channel creation.

Besides channel compactness, the boundary smoothness also affects the feasibility of accurately constructing the channel. Balasubramanian et al. [29]’s study introduced the spectral arc length $\mu_{sa}$ for smoothness assessment. However, this metric is less suitable for three-dimensional boundaries. Given that the surgical channel is primarily defined by the target points and associated entry points, the contours of feasible entry points on the control plane $B_{tS}$ determine the smoothness of the entire channel boundary. Thus, this evaluation can be conducted on the two-dimensional control plane:

(15) $$\mu_{sa}\left(C\right)=\mu_{sa}\left(L^{\prime }\left(1-\prod_{t\in T}\left(1-B_{tS}\right)\right)\right)$$

where $L^{\prime }\left(\;\cdot \;\right)$ represents the first derivative of the contour of a two-dimensional region.

By combining these two evaluations, the constructability of the planned surgical channel can be determined:

(16) $$Q_{c}\left(C_{mill}\right)=-\left(\mu_{gsc}\left(C_{mill}\right)/\mu_{gsc}\left(C_{mill}^{0}\right)\right)\;\cdot \;e^{\mu_{sa}\left(C_{mill}\right)}$$

Subsequently, the NSGA-III genetic optimization algorithm [30,31] was employed to iteratively refine the milling area, aiming for enhanced accessibility, safety, and constructability. Two solutions were selected from the Pareto frontier using the compromise programming algorithm [32] and the pseudo-weight algorithm [33]. Finally, the solution with the maximum $\mu_{gsc}\left(C\right)$ was chosen as the final result.

### 3.5. Automated Planning Acceleration

**Figure 6:**
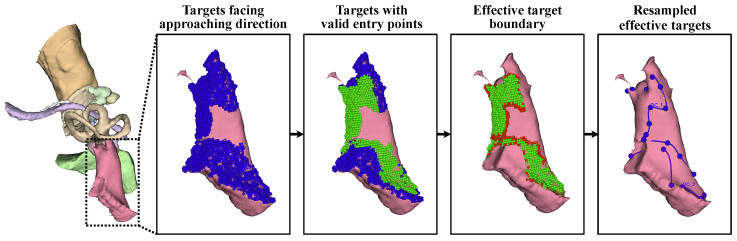
Effective surgical target contour extraction for accelerated computing while preserving the validity of the surgical channel. The most critical entry points were reserved to ensure the accessibility of the whole surgery target while reducing the time consumption. Blue points represent targets facing approaching direction, green points represent targets with valid entry points, red points represent effective target boundary, blue dot dash line represent resampled effective targets.

Although the proposed volumetric area modeling methods greatly simplify the definition space of the target milling area and make it compatible with optimization algorithms, the need to iterate over entry points K and surgical targets T, along with calculating the distances between the surgical tools and three-dimensional critical structures at a high spatial resolution, results in substantial time consumption. Therefore, this section introduces several methods (see Figure 6) aimed at accelerating the automated planning procedure.

**Symbolic Distance Field (SDF) of critical structures:** In generating the maximum milling area, substantial computation was required to determine the distances between the surgical tools and the surrounding critical structures. Due to the high resolution of the critical structures and the dense spatial distribution of the distance queries, neither point-by-point iteration over structures H nor k-d tree searches across the tool trajectories proved efficient enough for this task. To address this, three-dimensional signed distance fields $\left\{u_{sdf}^{\left(i\right)}\right\}$ were precomputed for each structure. In subsequent calculations, SDF lookup was employed to replace time-intensive nearest distance or distance calculations, and GPU acceleration was utilized to enhance the efficiency.

**Early termination strategy of the traversal process:** When calculating the valid entry points K(t) for each entry point, the contribution of a new effective entry point k∈K to the surgical channel $C_{mill}^{\left(0\right)}\left(t\right)$ gradually decreases throughout the iteration procedure. Moreover, the physical size of the surgical tool is much larger than the spatial resolution of the entry points. Therefore, the contribution of the new traversed entry point *k* was calculated:

(17) $$G_{t}\left(k\right)=\frac{C_{mill,t}\left({\left\{k\right\}}_{last}+k\right)-C_{mill,t}\left({\left\{k\right\}}_{last}\right)}{d\left(k,t\right)\;\cdot \;\pi r^{2}}$$

When $G_{t}$ consistently falls below $5\times 10^{-4}$, it is assumed that the maximum surgical channel has nearly been established, and the iteration process is terminated. After projecting K(t) onto the control plane for $B_{t,S}$, the pixelized effective area was processed using a morphological closing operation using a kernel of the physical size *r* to ensure continuity and integrity.

**Control of the target area’s resolution and effective target extraction:** During surgery, only the surface of the target needs to be exposed to allow access to the target, and the flexibility of the surgical target allows surgeons to reach the full target even with partial surface exposure. Meanwhile, the large number of target points increases the number of optimization parameters, slowing the convergence process. Therefore, target points were filtered to retain only effective points on the surface.

First, target points that were not oriented toward the approach direction were excluded. For each target t∈T, the normal vector at this point, $n_{t}$, was estimated. For any potential entry point k∈K, the angle between the normal and the tool trajectory was calculated as $\theta \left(t,k\right)=\arccos\left(n_{t}\;\cdot \;\vec{tk}\right)$. The count of *k* satisfying θ(t,k)<π/2 was recorded as $r_{t}$. A target point *t* was deemed effective if it could be accessed from multiple entry points: $r_{t}>\mathrm{mean}\left(r\right),\;r_{t}>0.05\times \mathrm{Num}\left(K\right)$. The resulting effective target points form $T_{\left(1\right)}$.

After that, the accessibility of each $t\in T_{\left(1\right)}$ was further verified under the constraints imposed by H. To expedite this process, K was resampled with a spatial resolution of 2r, and the iteration terminated once any valid *k* was identified. This step further refined the target points into $T_{\left(2\right)}$.

Given the convexity of the surgical target and the spatial distribution of the critical structures, $T_{\left(2\right)}$ is fully accessible if all the points on its boundary are reachable. Therefore, the target area $T_{\left(2\right)}$ was further compressed to its contour line $T_{\left(3\right)}$.

Finally, $T_{\left(3\right)}$ was arranged in clockwise order along the boundary of $T_{\left(2\right)}$ as a closed curve and downsampled to $T_{\left(4\right)}$ using *r* as the spatial interval to ensure a partial overlap between adjacent access trajectories.

By refining the target points, approximately 20 target points remained for subsequent optimization, while the validity of the planned surgical channel could still be ensured. With 8 parameters per control function $\sigma_{t}$, only about 160 parameters were required to control the irregular volumetric milling area.

## 4. Experiments and Results

To comprehensively evaluate the proposed automated milling area planning algorithm, an experiment was conducted on six clinical CT volumes, comparing the results with those of manual planning. The dataset included three left-ear and three right-ear cases. Acoustic neuromas were used as the surgical targets in the planning process, with eight parameters controlling the maximum milling area for each target point. Additionally, the safe distances $d_{risk}$ were set to 1.5 mm for the sinus dura, 2.5 mm for the facial nerve, and 0.5 mm for the other critical structures, based on guidance from an experienced surgeon. The time consumption and evaluation metrics for the surgical channel were recorded.

### 4.1. Time Consumption

**Figure 7:**
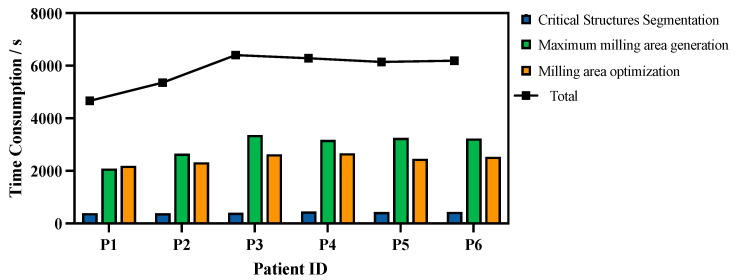
Time consumption in each step of the algorithm on different data.

The time consumption was measured on a graphics workstation with an Intel Core i9-13900K CPU, an NVIDIA GeForce RTX 4090 GPU, and Ubuntu 20.04.1 LTS. Multi-objective milling area optimization was implemented using PyMOO [34], while the parallelizable components of the algorithm were implemented using CuPy [35] for GPU acceleration. The time consumption for each step of the algorithm is shown in Figure 7.

On average, 5840.1 ± 279.9 s was required for the entire planning procedure. Of this duration, 416.9 ± 10.7 s was spent on critical structure segmentation, 2958.5 ± 203.1 s on maximum milling area generation, and 2464.7 ± 74.4 s on milling area optimization.

### 4.2. Evaluation Metrics for Each Step

**Figure 8:**
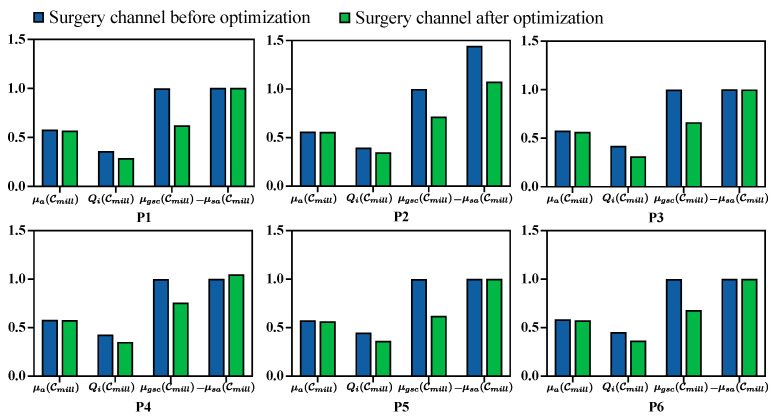
Surgical channel evaluations for each CT volume before and after optimization. The indicators include surgical target accessibility $\mu_{a}\left(C_{mill}\right)$, structure damage $Q_{i}\left(C_{mill}\right)$, global spatial compactness $\mu_{gsc}\left(C_{mill}\right)$, and boundary smoothness $\mu_{sa}\left(C_{mill}\right)$. The vertical axis represents normalized indicators. For $\mu_{a}\left(C_{mill}\right)$, $\mu_{gsc}\left(C_{mill}\right)$, and $\mu_{sa}\left(C_{mill}\right)$, the higher the better; for $Q_{i}\left(C_{mill}\right)$, the lower the better.

**Figure 9:**
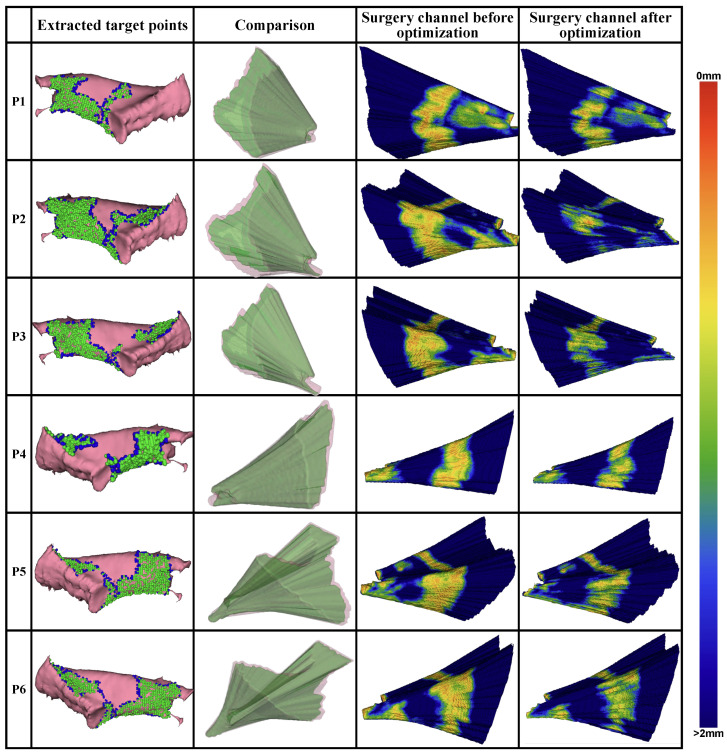
Visualization of each step in the automatic planning procedure, including the optimal operation area extracted from the surgery target and the distance from the milling area boundaries to the surrounding critical structures before and after optimization.

The quantitative evaluation for each metric before and after milling area optimization is presented in Figure 8. Additionally, the generated target points for optimization, the planned milling volume, and the distance distribution from the volume surface to the critical structures are visualized in Figure 9.

In general, target points oriented toward the approach direction of the surgical operations and the boundary used for the milling area were extracted from the labeled surgical target. The distribution of the distance to the surrounding critical structures on the surface of the planned surgical channel before and after optimization shows a substantial reduction in the areas near the critical structures after optimization. Quantitatively, the average number of voxels within a 1 mm distance from the critical structures decreased by 71.3% across all CT volumes.

The quantitative evaluation metrics for the planned surgical channel, including surgical target accessibility $\mu_{a}\left(C_{mill}\right)$, structure damage $Q_{i}\left(C_{mill}\right)$, global spatial compactness $\mu_{gsc}\left(C_{mill}\right)$, and boundary smoothness $\mu_{sa}\left(C_{mill}\right)$, were recorded before and after optimization (see Figure 8). Generally, the planned milling area is better with a higher value for $\mu_{a}$, a lower value for $Q_{i}$, and a higher value for $\mu_{sa}$, while $\mu_{gsc}$ is better when it is higher under the same milling volume and will increase as the milling volume increases. Following the optimization process, $\mu_{a}\left(C_{mill}\right)$, $Q_{i}\left(C_{mill}\right)$, and $\mu_{sa}\left(C_{mill}\right)$ improved by 1.4%, 18.9%, and 3.5%, respectively, while $\mu_{gsc}\left(C_{mill}\right)$ decreased by 32.3%. This reduction in $\mu_{gsc}\left(C_{mill}\right)$ is proportional to the volume size. During optimization, the milling area was minimized to reduce structural damage, which consequently lowered $\mu_{gsc}\left(C_{mill}\right)$.

### 4.3. Comparison with Manual Planning

To verify the feasibility of the milling area planned using the proposed method further, we compared the planning results against those from manual planning performed by surgeons. An experienced surgeon was recruited to manually plan the milling area based on CT images. The planning procedure was conducted using 3D Slicer software with the segmentation editor. To ensure a fair comparison, the surgeon did not have access to the automated planning results, while the same critical structure segmentations were provided. Additionally, since the surgeon primarily used a slice-by-slice planning strategy, a Gaussian filter with a radius of 0.25 mm was applied to the planned results to fill any gaps in the volume.

**Figure 10:**
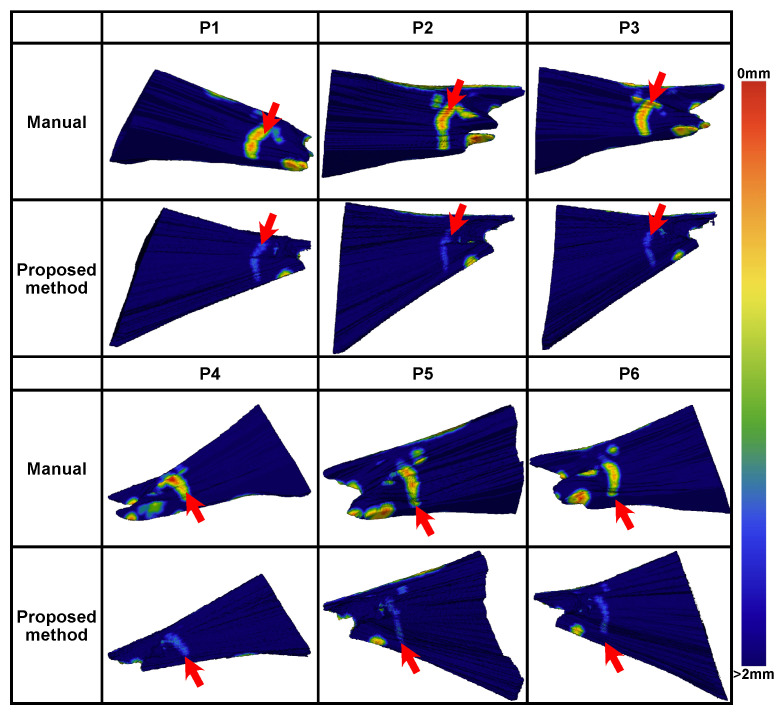
Comparisons of planned milling area and distances to critical structures using proposed planning algorithm and manual planning.

A comparison between manual and automated planning is shown in Figure 10. Generally, the automated planning effectively avoided high-risk areas within 1 mm of the critical structures. In each case, high-risk areas within 0.5 mm from the critical structures existed for the manual planning.

**Figure 11:**
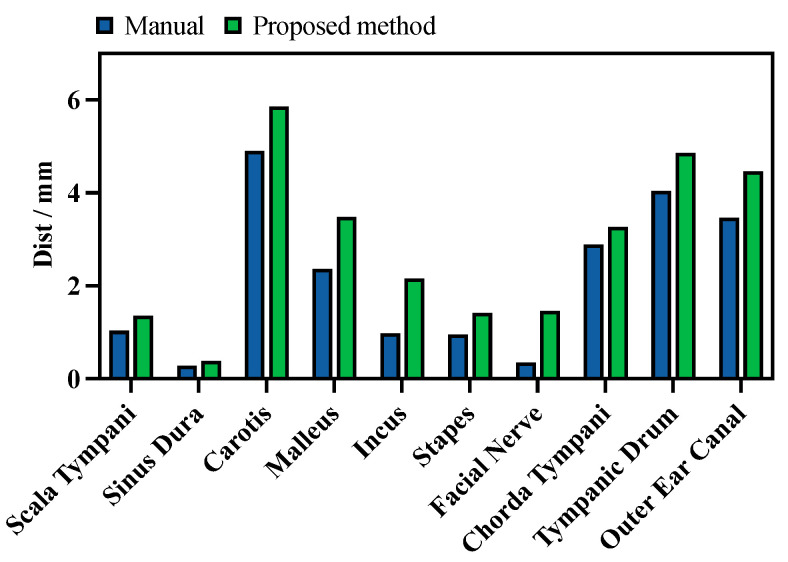
Average minimum distance from planned milling area to each critical structure under automatic and manual planning (the higher, the better).

The minimal distances from the planned milling volume to each critical structure are shown in Figure 11, averaged across all six datasets. Compared to the manual planning results, the minimal distances were increased by 0.94 ± 1.24 mm, 0.80 ± 0.43 mm, 0.90 ± 0.41 mm, 0.52 ± 0.48 mm, 0.79 ± 0.64 mm, and 0.51 ± 0.25 mm for the scala tympani, the sinus dura, the carotis, the malleus, the incus, the stapes, the facial nerve, the chorda tympani, and the outer ear canal, respectively. Average increases of 72.0%, 86.3%, 112.1%, 56.5%, 83.7%, and 50.6% were achieved.

**Figure 12:**
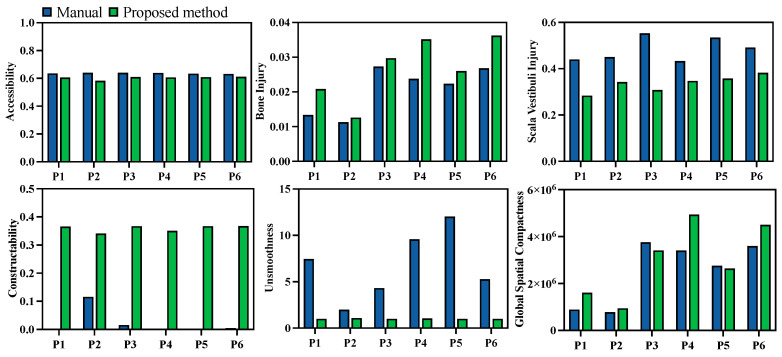
Comparison of automatic and manual planning in terms of accessibility, bone injury, scala vestibuli injury, boundary smoothness, and global spatial compactness. For accessibility, constructability, and global spatial compactness, the higher, the better. For bone injury, scala vestibuli injury, and lack of smoothness, the lower, the better.

Quantitative evaluations of both the manual and automated planning results were also compared. Unlike the previous comparison of the results before and after multi-objective optimization, the injury evaluation $Q_{i}$ was divided into two categories—damage to the temporal bone and damage to the scala vestibuli—since damage to the scala vestibuli is a critical metric in clinical cases. The evaluation results are presented in Figure 12. Comparatively, damage to the scala vestibuli was reduced by 29.8%, while the milling volume increased by 29.3% with the automated algorithm. Additionally, the channel boundary smoothness was improved by 78.3%, and the global spatial compactness was improved by 26.4%. The corresponding constructability of the milling area was improved by more than 2.95 times in all cases. The performance in terms of target accessibility was similar for manual and automated planning.

To further compare manual and automated planning, an experienced surgeon was recruited to rate both planning results. This surgeon was different from the one who performed the manual planning. To ensure fairness, the surgeon was not informed about whether each plan was manual or automated. All 12 planned results were randomized in sequence for evaluation, and the critical structure segmentations were provided for reference. During evaluation, the surgeon could freely view each result on both 2D slices and 3D volumes using 3D Slicer. After examining each result, the surgeon completed a questionnaire with three questions focusing on injury risk, constructability, and accessibility:

- Q1: Is the planned surgical channel likely to damage vascular, nerve, or any other risk structures during bone milling?
- Q2: Is it possible to successfully construct the planned surgical channel using common neurosurgical procedures such as milling?
- Q3: After the removal of the corresponding bone tissue, is it possible to successfully achieve the surgical target?

**Figure 13:**
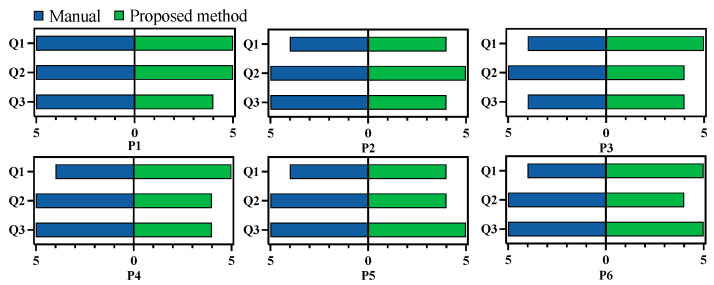
Subjective evaluation of automated and manual planned milling areas.

Each question was rated on a scale from 1 to 5, with 1 being the lowest and 5 the highest score. The results of this evaluation are presented in Figure 13. On average, the manual planning results scored 4.2, 5, and 4.8 for the three questions, while the automated results scored 4.7, 4.3, and 4.3. The surgeon noted that manual planning posed a higher risk to the surrounding structures, potentially due to less comprehensive consideration of the spatial relationship between the milling area and the adjacent structures. Additionally, the surgeon reported that the automated planning results were clinically feasible.

## 5. Discussion

In this paper, we propose an automated planning method utilizing an optimization method to obtain a safe and executable surgical plan for the milling areas in mastoidectomy. To accomplish this, a template-based approach was employed to segment high-resolution critical structures surrounding the target operation area, using two templates for segmentation via a global-to-local registration process. Following segmentation, the maximum milling area was defined by simulating the access to the target during the surgical procedure. Re-categorization was then applied to the elements within the maximum milling area, with potential entry points projected onto a control plane to simplify the complex expressions caused by the irregular structural surfaces. Next, a set of control functions was introduced to deform the maximum milling area into a subset in a parameterized manner. Finally, the milling area’s constructability, the post-milling accessibility of the surgical target, and the injury risk to the patient were quantitatively evaluated. Multi-objective optimization was then employed to derive an optimal milling plan that met both the safety and feasibility requirements.

In general, the proposed method represents the irregular volumetric area as a combination of multiple cylinders aligned with the physical dimensions of the surgical tools, extending from the entry points to the target. The access trajectories are organized by each target point and are deformed on the entry side, ensuring the effectiveness of each spatial element, even within the deformed surgical channel. Additionally, the definition of the control functions with control points and interpolation preserves the volume’s smoothness. By adopting the maximum milling area as a deformation reference, spatial constraints are transformed into numerical limitations on the control parameters, making the irregular volumetric area suitable for optimization algorithms. Compared to previous works aiming at volumetric milling area planning, Popovic et al. [21] neglected the thickness of the skull for heuristic generation, while McBrayer et al. [17], Aghdasi et al. [22], and Rajesh et al. [2] pre-planned the milling area on an atlas and mapped it to new CT data for patient-specific milling areas. To our knowledge, our work is the first to achieve successful adoption of optimization methods in this procedure for more feasible milling area planning.

As a result, improved using the computation acceleration strategies adopted in this study, the proposed method could fully and automatically plan the milling area within 5840.1 ± 685.7 s. Due to the long time interval between CT scanning and the conduction of surgery, this time consumption already satisfies the clinical requirements. Moreover, it is still possible to improve the algorithm towards its real-time application. The time consumption of the current algorithm mainly lies in the generation of the maximum milling area and the multi-objective optimization. In both procedures, the spatial relationships between different targets need to be repetitively calculated, leading to a considerable amount of parallel computation, and might be improved by adopting a more powerful GPU. Moreover, the current implementation was based on Python, where a large overhead exists. Implementing the same method using C++ and CUDA, with careful organization to reduce repetitive calculations and the overhead, could significantly reduce the time consumption.

Compared to the manual planning results, our proposed method reduced the damage to the scala vestibuli by 29.8% and increased the distance from the milling area to the surrounding critical structures by percentages ranging from 50.6% to 112.1%, significantly enhancing the safety of the milling procedure. Additionally, the boundary smoothness was improved by 78.3% and global spatial compactness by 29.8% when automated planning was used, making the planned milling area easier to construct during surgery. As the surgeon primarily adopted a slice-by-slice planning strategy, comprehensive consideration of the spatial relationship between different slices was lacking, leading to the reduced constructability of the planned volume. In contrast, our method generates a volume fully controlled by the entry and target points, theoretically ensuring that each spatial element is accessible during milling operations. In addition to the quantitative evaluation, a subjective assessment by an experienced surgeon was included. Although the automated plan requires more bone removal, the surgeon observed that the planned volumes maintained a safer distance from the surrounding structures and were feasible for real surgical cases. In most acoustic neuroma cases, the critical structures surrounding the lesion area remain unaffected; therefore, the generalizability of the proposed method will not be influenced by the level of the tumor lesion. However, evaluation on a more diverse dataset is still needed in future work.

Although this paper primarily focuses on mastoidectomy, the volumetric area modeling method is not limited to this clinical case. This approach—using entry and target points for volume representation and employing a maximum area and parameterization strategy for deformation—could also be applied in other situations where complex shapes and constraints need to be quantitatively represented.

Meanwhile, there are several limitations regarding the proposed method that need to be addressed in future studies. During the implementation of the proposed method, the surgical tool was modelized using a cylinder to simplify the calculation. Certain limitation might be raised due to this setup. For most neurosurgical tools, the three-dimensional spatial constraints can be simplified using a cylinder bounding box. However, a volumetric representation of the surgical tool might be needed when a more accurate description of the tool’s shape is needed, which would potentially increase the computational consumption of the proposed method. A way to consider the spatial constraints in such cases remains an open question. Moreover, the proposed method is also dependent on high-quality preoperative CT data and segmentation labels. How to provide a precise distribution of the critical structures when the imaging quality is limited also needs to be discussed. A way to integrate automatic planning into the clinical procedure is also a critical problem. Surgeons need to be aware of the possible risks of the milling plan and understand the evaluation metrics during automatic planning, and a mechanism to integrate the surgeons’ clinical experience during planning will be needed to ensure the safety of the surgery.

## 6. Conclusions

This paper introduced a modeling method for irregular volumetric areas, enabling automated planning of the milling area in acoustic neuroma surgery. By pre-generating the maximum milling area based on the spatial constraints imposed by the surrounding critical structures, the milling area was fully parameterized using several control functions and a deformation process. Additionally, the complex spatial constraints were translated into numerical limitations on the parameters. The milling area was evaluated using multiple metrics, including accessibility, constructability, and injury, while multi-objective optimization was employed to achieve the optimal planning results. Overall, the proposed planning procedure was able to generate a milling plan within a reasonable time, while the risks during bone milling were significantly reduced. The automatically planned milling areas also demonstrated smoother boundaries, enhancing their feasibility for real clinical applications. This modeling approach is applicable not only to acoustic neuroma surgery but also to other cases where complex relationships between a volumetric target and the surrounding structures need to be considered.

## Data Availability

The data that support the findings of this study are available from the corresponding author upon reasonable request.
